# Supplementary material for: Ecological similarities and dissimilarities between donor and recipient regions shape global plant naturalizations
Source: Nat Commun. 2025 Nov 25;16:10485. doi: 10.1038/s41467-025-65455-y (PMC12647834; doi:10.1038/s41467-025-65455-y)
Supplement: Supplementary file 1 — Supplementary Information [file 41467_2025_65455_MOESM1_ESM.pdf]

Supplemental Information for

Ecological similarities and dissimilarities between donor and recipient regions shape global plant naturalizations

Shu-ya Fan, Trevor S. Fristoe, Shao-peng Li*, Patrick Weigelt, Holger Kreft, Wayne Dawson, Marten Winter, Petr Pyšek, Jan Pergl, Franz Essl, Amy J.S. Davis & Mark van Kleunen

*Correspondence to: spli@des.ecnu.edu.cn

Supplementary table 1 | Normalization methods applied and the contributions of 19 bioclimatic variables to the first two axes of a principal component analysis (PCA).

Bioclimatic variables	Transformation function	D value of Kolmogorov-Smirnov test	Loadings	
			PC_{Temp}	PC_{Prec}
Annual mean temperature	inverse log	0.09	0.30	-0.17
Annual precipitation	boxcox	0.02	0.21	0.30
Isothermality	boxcox	0.06	0.31	-0.05
Max temperature of warmest month	boxcox	0.05	0.20	-0.27
Mean diurnal range	boxcox	0.03	-0.01	-0.28
Mean temperature of coldest quarter	inverse log	0.08	0.33	-0.08
Mean temperature of driest quarter	inverse log	0.08	0.27	-0.16
Mean temperature of warmest quarter	inverse log	0.05	0.21	-0.27
Mean temperature of wettest quarter	inverse log	0.07	0.23	-0.17
Min temperature of coldest month	inverse log	0.07	0.33	-0.03
Precipitation of coldest quarter	boxcox	0.03	0.11	0.30
Precipitation of driest month	boxcox	0.10	0.04	0.35
Precipitation of driest quarter	boxcox	0.05	0.06	0.36
Precipitation of warmest quarter	sqrt	0.03	0.14	0.29
Precipitation of wettest month	boxcox	0.03	0.23	0.23
Precipitation of wettest quarter	boxcox	0.03	0.22	0.24
Precipitation seasonality	sqrt	0.03	0.05	-0.23
Temperature annual range	cube root	0.04	-0.31	-0.07
Temperature seasonality	boxcox	0.05	-0.32	-0.03
Eigenvalue			8.44	6.32
Proportion of variance			44.42	33.25
Cumulative proportion			44.42	77.67

Columns from left to right: bioclimatic variable name; transformation function yielding the closest approximation to a normal distribution; D value from the Kolmogorov-Smirnov test for the selected transformation; and loadings on the first two PCA axes.

Supplementary table 2 | Standardized coefficients of linear and quadratic terms for ecological distances between donor and recipient regions in predicting naturalization probability.

Variable	Term	Std. coefficient	Std. error	*z* value	*P* value	CI_low	CI_high
PC_{Temp} distance	Linear term (β_1)	0.89	0.03	26.59	<0.001	0.82	0.96
	Quadratic term (β_2)	-1.32	0.01	-152.21	<0.001	-1.34	-1.30
PC_{Prec} distance	Linear term (β_1)	0.53	0.03	20.22	<0.001	0.48	0.58
	Quadratic term (β_2)	-0.20	0.00	-47.86	<0.001	-0.21	-0.19
Human modification distance	Linear term (β_1)	0.39	0.03	12.79	<0.001	0.33	0.45
	Quadratic term (β_2)	-0.09	0.00	-19.70	<0.001	-0.10	-0.08
Native flora phylogenetic diversity distance	Linear term (β_1)	-1.82	0.04	-48.46	<0.001	-1.90	-1.75
	Quadratic term (β_2)	-0.03	0.01	-4.52	<0.001	-0.04	-0.02
Floristic phylogenetic dissimilarity	Linear term (β_1)	-0.45	0.03	-16.28	<0.001	-0.50	-0.40
	Quadratic term (β_2)	-0.07	0.00	-16.34	<0.001	-0.08	-0.06
Geographical distance	Linear term (β_1)	0.95	0.02	43.10	<0.001	0.91	1.00
	Quadratic term (β_2)	-0.12	0.00	-29.70	<0.001	-0.13	-0.11

Columns from left to right: variable name; term type (linear or quadratic); standardized coefficient (β); standard error (SE); Wald z value; P value; lower and upper bounds of the 95% confidence interval (CI). All tests were two-sided Wald z-tests; no adjustments for multiple comparisons were applied.

Supplementary table 3 | Mean variable importance of each ecological distance in predicting naturalization probability across 999 bootstrap samples.

Variable	R^2 type	Individual contribution	Relative importance (%)
PC_{Temp} distance	delta	0.09	63.71
	theoretical	0.39	56.21
PC_{Prec} distance	delta	0.01	10.46
	theoretical	0.09	13.53
Human modification distance	delta	0.00	3.23
	theoretical	0.03	4.29
Native flora phylogenetic diversity distance	delta	0.03	18.86
	theoretical	0.14	20.21
Floristic phylogenetic dissimilarity	delta	0.00	1.66
	theoretical	0.02	3.33
Geographical distance	delta	0.00	2.07
	theoretical	0.02	2.43
Model marginal R^2	delta	0.14	
	theoretical	0.70	
Model conditional R^2	delta	0.16	
	theoretical	0.78	

Columns from left to right: variable name; R^2 type (delta or theoretical); mean individual contribution across 999 bootstrap samples; mean percentage contribution across 999 bootstrap samples.

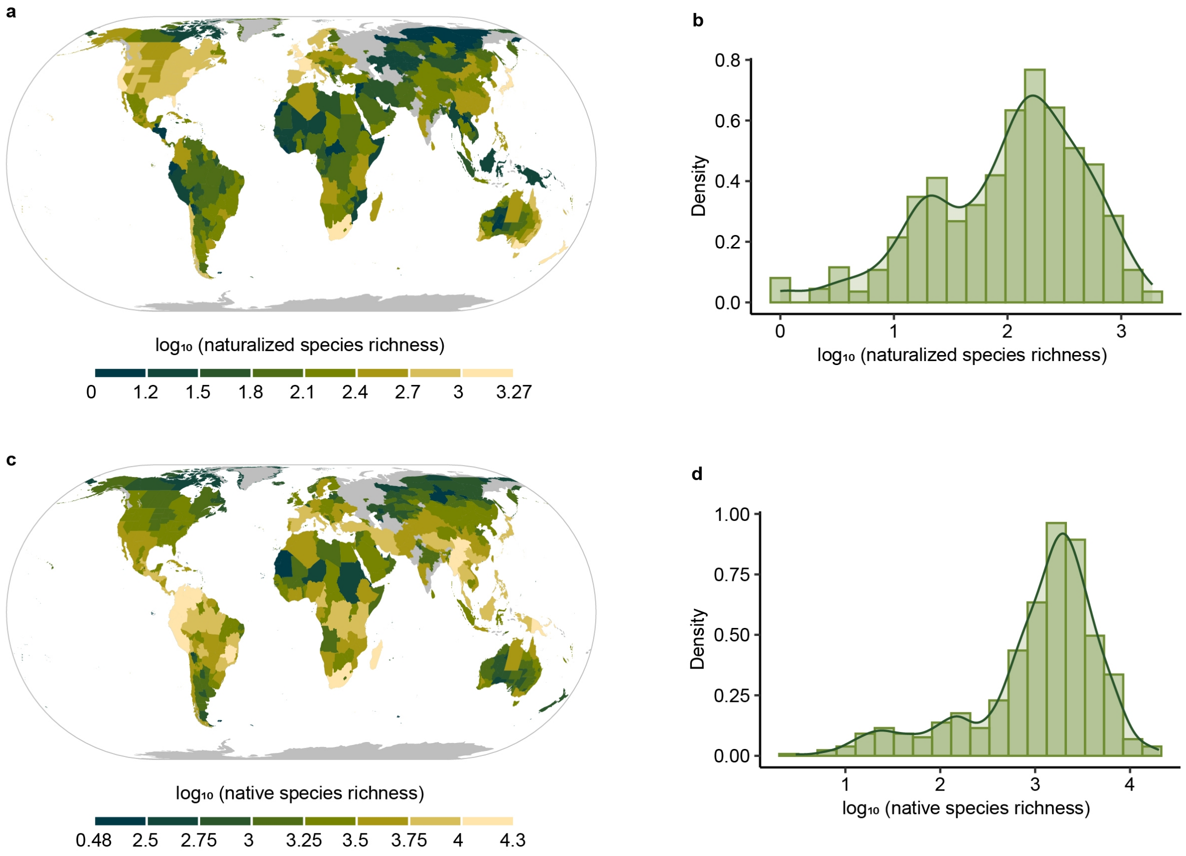

Supplementary Fig. 1 | Maps and histograms of naturalized and native species richness. Global maps showing the number of naturalized species (**a**) and the number of native species (**c**) across the 650 regions included in our study. Gray areas indicate regions without data. The histograms display the density distribution of naturalized (**b**) and native (**d**) species richness, respectively. Species richness was log10-transformed. Polygons were generated from the GloNAF database, with country boundaries obtained from Natural Earth (https://www.naturalearthdata.com/). Source data are provided as a Source Data file.

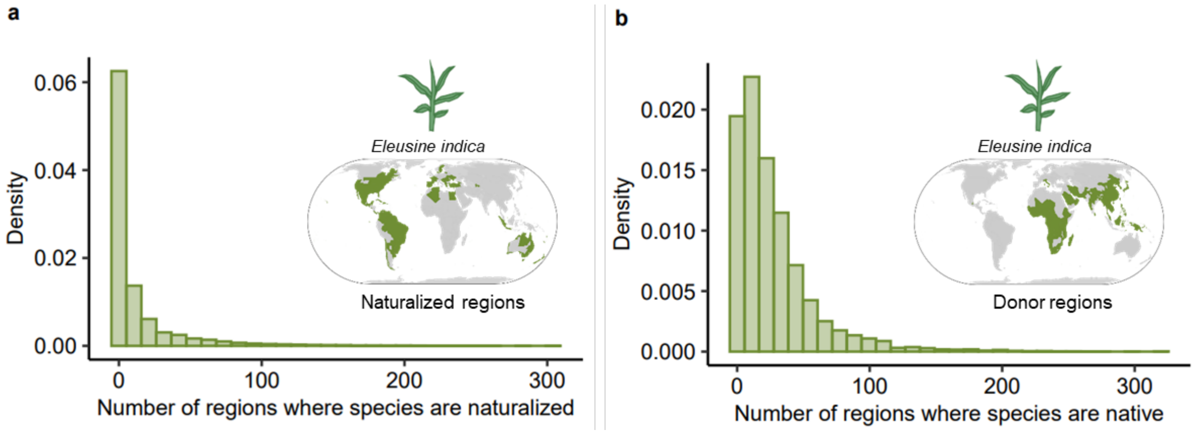

Supplementary Fig. 2 | Density distributions of the number of regions where each species is naturalized (a) and native (b). Insets show, as an example, the global distribution for *Eleusine indica*, with 258 naturalized regions and 115 native regions. Green areas represent regions where the species is naturalized or native. Polygons were generated from the GloNAF database, with country boundaries obtained from Natural Earth (https://www.naturalearthdata.com/). Source data are provided as a Source Data file.

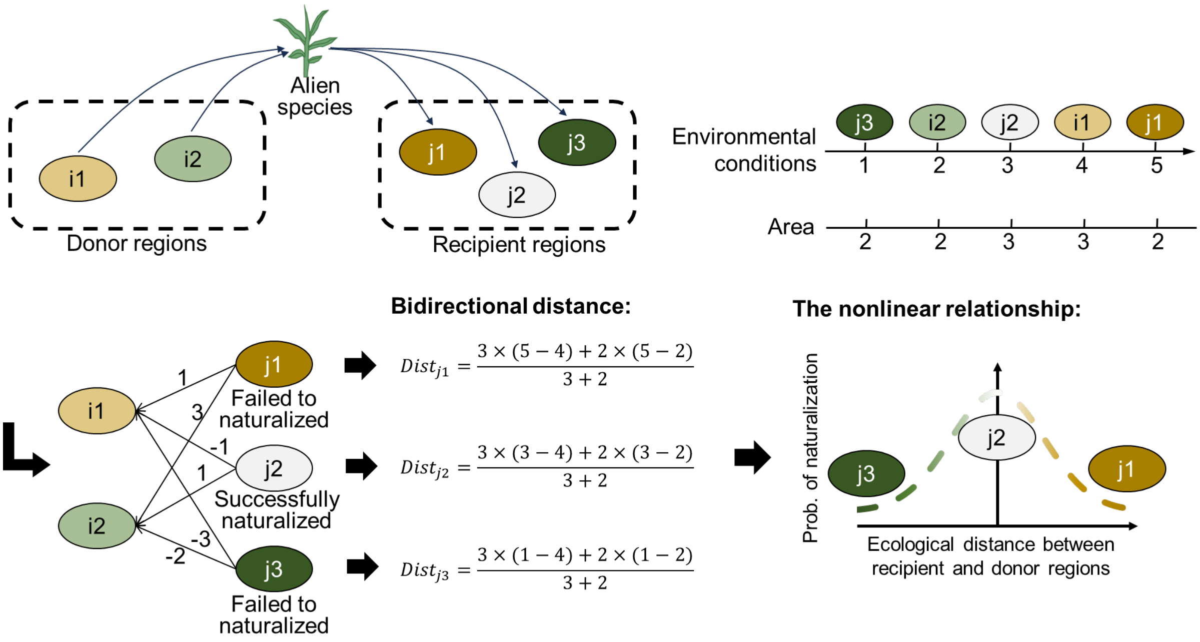

Supplementary Fig. 3 | A schematic diagram illustrating the calculation of bidirectional ecological distance metrics between recipient and donor regions for an alien species. i1 and i2 represent the donor (native) regions of an alien species, and j1–j3 represent the potential recipient regions. The colour gradient of the regions, from green to yellow, indicates environmental conditions ranging from lower to higher values. Arrows denote the direction of species transfers from donor to recipient regions.

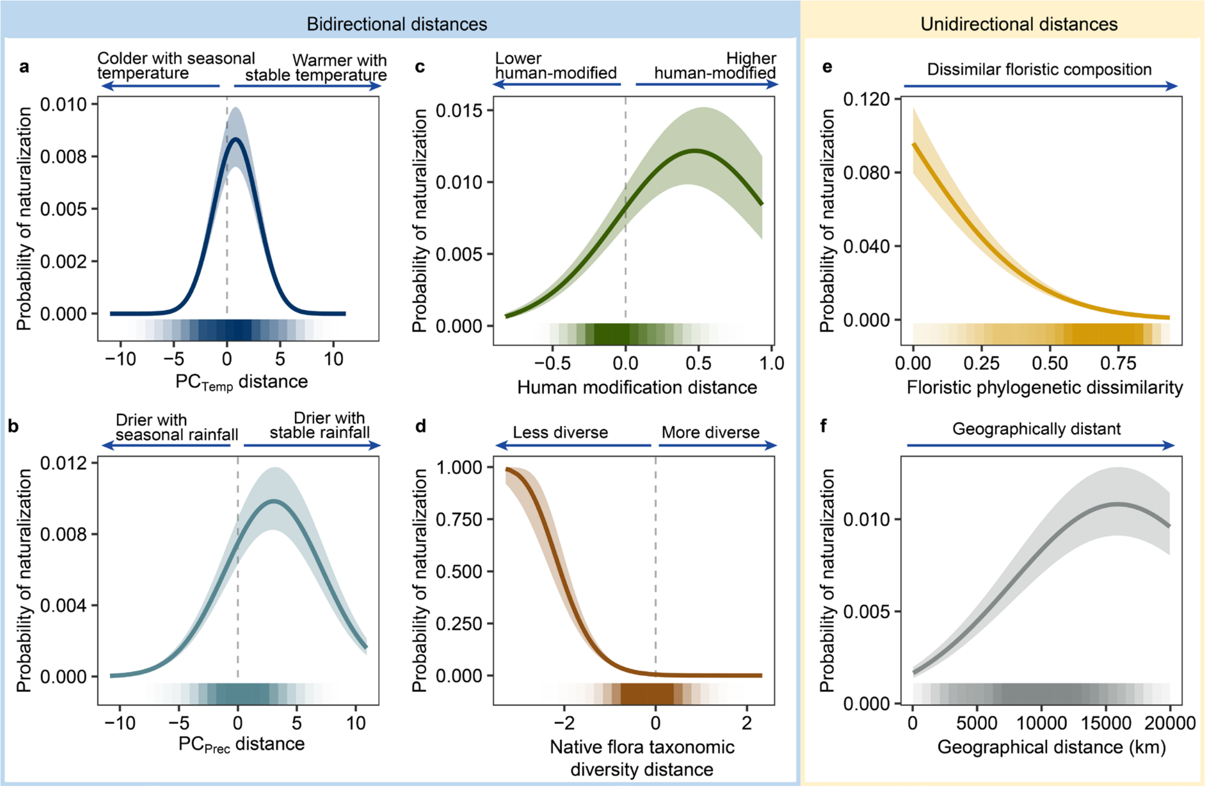

Supplementary Fig. 4 | Partial relationships between naturalization probability and ecological distances between recipient and donor regions with native flora taxonomic diversity distance included as a predictor variable instead of native flora phylogenetic diversity distance. The multivariate generalized linear mixed-effects model, based on 6,931,789 observations derived from the naturalization of 11,604 alien plant species across 650 regions, included linear and quadratic terms for each of the six ecological distances (**a-f**). The solid lines represent the predicted mean (model fit), and the shaded areas denote the 95% confidence intervals of these predictions. The bar below each plot indicates the number of data points in each bin where the predictor variable was divided into 30 segments, with darker shades indicating more data points within each bin. Note that the y-axis scales differ across panels to reflect variations in data range. Source data are provided as a Source Data file.

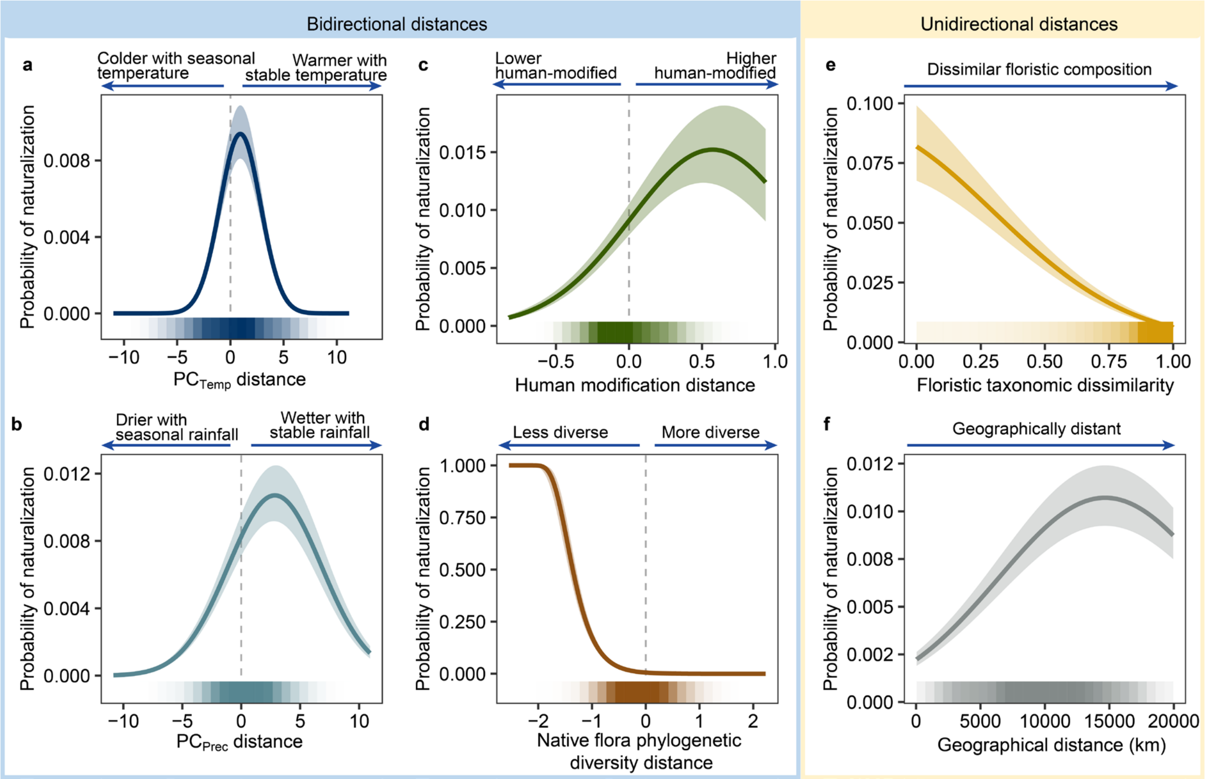

Supplementary Fig. 5 | Partial relationships between naturalization probability and ecological distances between recipient and donor regions with floristic taxonomic dissimilarity included as a predictor variable instead of floristic phylogenetic dissimilarity. The multivariate generalized linear mixed-effects model, based on 6,931,789 observations derived from the naturalization of 11,604 alien plant species across 650 regions, included linear and quadratic terms for each of the six ecological distances (**a**-**f**). The solid lines represent the predicted mean (model fit), and the shaded areas denote the 95% confidence intervals of these predictions. The bar below each plot indicates the number of data points in each bin where the predictor variable was divided into 30 segments, with darker shades indicating more data points within each bin. Note that the y-axis scales differ across panels to reflect variations in data range. Source data are provided as a Source Data file.

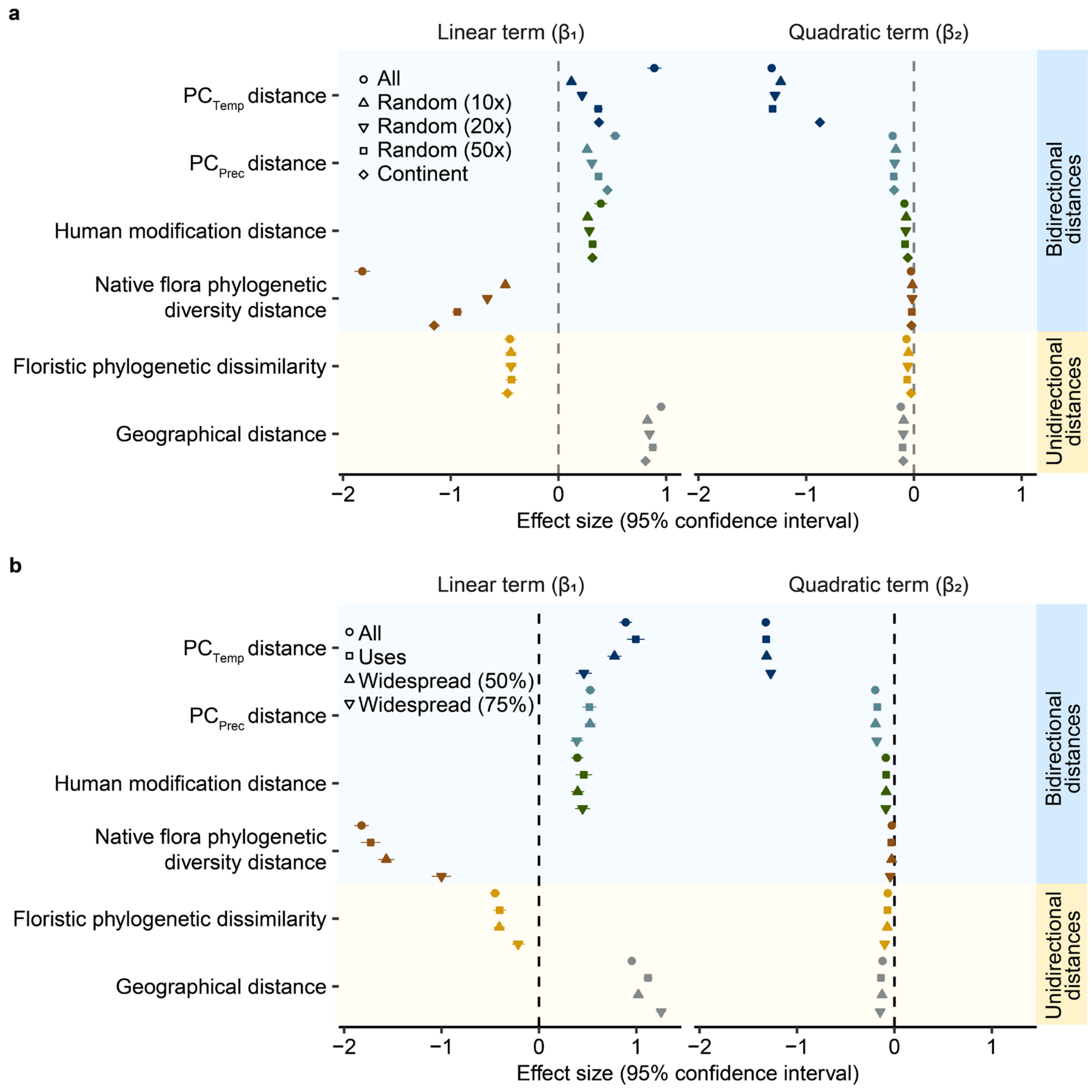

Supplementary Fig. 6 | Standardized coefficients of the linear and quadratic terms of the ecological distances between recipient and donor regions on naturalization probability across different data subsets. In **a**, different types of points represent standardized coefficients based on different sets of potential recipient regions: all non-native regions (All; as shown in Fig. 2); regions randomly selected from all non-native regions where naturalization has not occurred, for 10 (Random 10×), 20 (Random 20×), or 50 (Random 50×) times the number of regions in which the species has successfully naturalized, combined with the already naturalized regions; all non-native regions within the continents where the species has naturalized in at least one region (Continent). In **b**, different types of points represent

standardized coefficients based on different subsets of naturalized species: all naturalized species (All; as shown in Fig. 2), the subset of naturalized species with known economic uses (Uses), and subsets of species widely naturalized across multiple regions, specifically those naturalized in at least 2 regions (50th percentile, Widespread (50%)) and those naturalized in at least 10 regions (75th percentile, Widespread (75%)). The standardized coefficients of the ecological distance metrics on naturalization probability for each data subset were estimated by a multivariate generalized linear mixed-effects model. Points represent the standardized coefficients of the linear and quadratic terms of each distance metric, and error bars indicate their 95% confidence intervals derived from model-based standard errors; in several cases, the intervals are narrower than the symbols and thus not visible. All standardized coefficients are significantly different from zero (all P values from two-sided Wald z-tests < 0.01). No adjustments for multiple comparisons were applied. Source data are provided as a Source Data file.

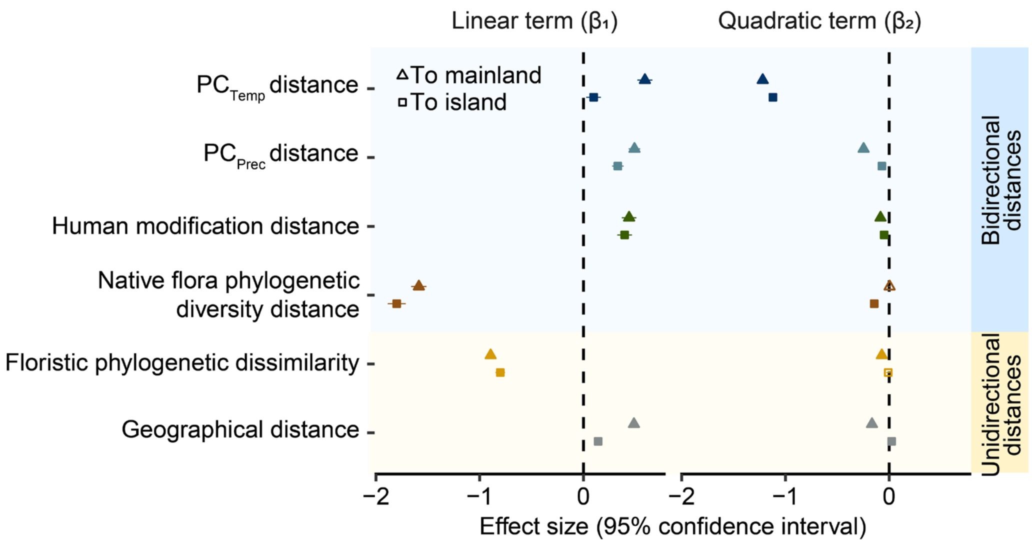

Supplementary Fig. 7 | Standardized coefficients of the linear and quadratic terms of ecological distances between recipient and donor regions on naturalization probability in mainland and island regions. Different types of points represent standardized coefficients based on species naturalizing in mainland and island regions. The standardized coefficients of the ecological distance metrics on naturalization probability for each data subset were estimated using a multivariate generalized linear mixed-effects model. Points represent the standardized coefficients of the linear and quadratic terms of each distance metric, and error bars indicate their 95% confidence intervals derived from model-based standard errors; in several cases, the intervals are narrower than the symbols and thus not visible. Solid points indicate variables with significant effect sizes (two-sided Wald z-tests: $P < 0.05$), while open points indicate non-significant effect sizes (two-sided Wald z-tests: $P \geqslant 0.05$). No adjustments for multiple comparisons were applied. Source data are provided as a Source Data file.

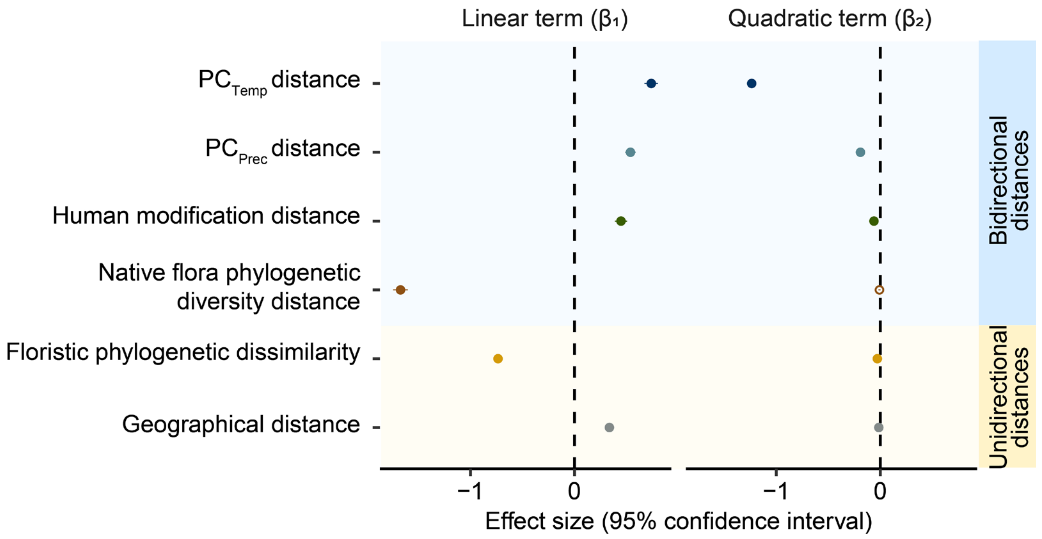

Supplementary Fig. 8 | Standardized coefficients of the linear and quadratic terms of ecological distances between recipient and donor regions on naturalization probability when species with conflicting status were classified as naturalized. The standardized coefficients of the ecological distance metrics on naturalization probability were estimated using a multivariate generalized linear mixed-effects model. Points represent the standardized coefficients of the linear and quadratic terms of each distance metric, and error bars indicate their 95% confidence intervals derived from model-based standard errors; in several cases, the intervals are narrower than the symbols and thus not visible. Solid points indicate variables with significant effect sizes (two-sided Wald *z*-tests: $P < 0.05$), while open points indicate non-significant effect sizes (two-sided Wald *z*-tests: $P \geqslant 0.05$). No adjustments for multiple comparisons were applied. Source data are provided as a Source Data file.

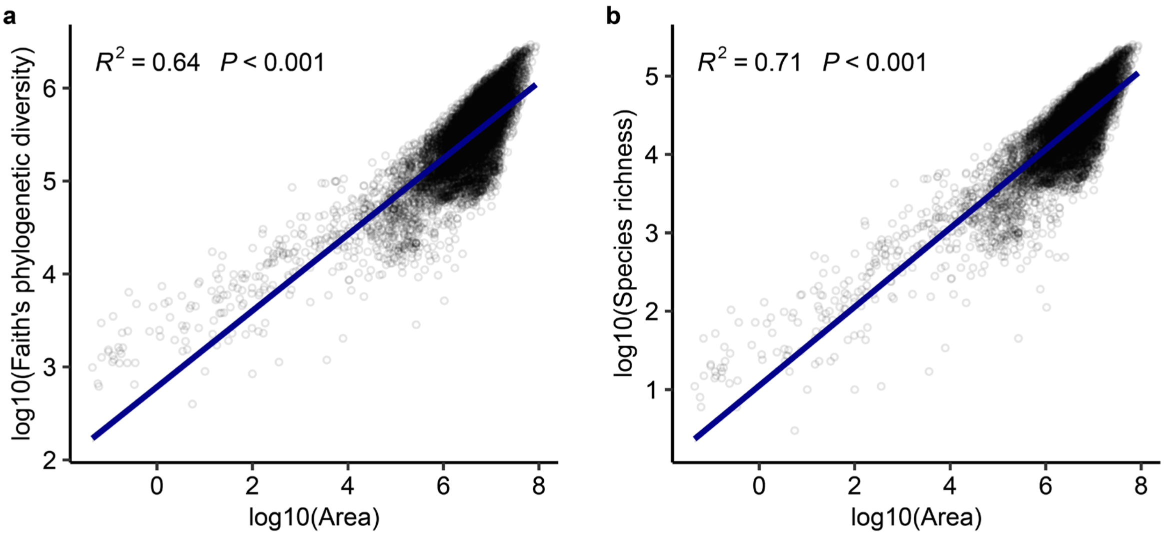

Supplementary Fig. 9 | The relationships between regional area and Faith's phylogenetic diversity (Faith's PD, a), as well as area and species richness (b) for regions and donor regions of each naturalized species, based on 11,604 alien plant species across 650 regions. The solid lines represent the fitted linear regressions, with gray shading indicating the 95% confidence intervals. For Faith's PD, the relationship is significant ($\beta = 0.41 \pm 0.003$ SE, $R^2 = 0.64$, $P < 0.001$), and for species richness, the relationship is also significant ($\beta = 0.50 \pm 0.003$ SE, $R^2 = 0.71$, $P < 0.001$). These regressions were used to generate area-corrected estimates of phylogenetic and taxonomic diversity (i.e., the residuals from the models). Note that area, Faith's PD, and species richness are log10-transformed. Source data are provided as a Source Data file.

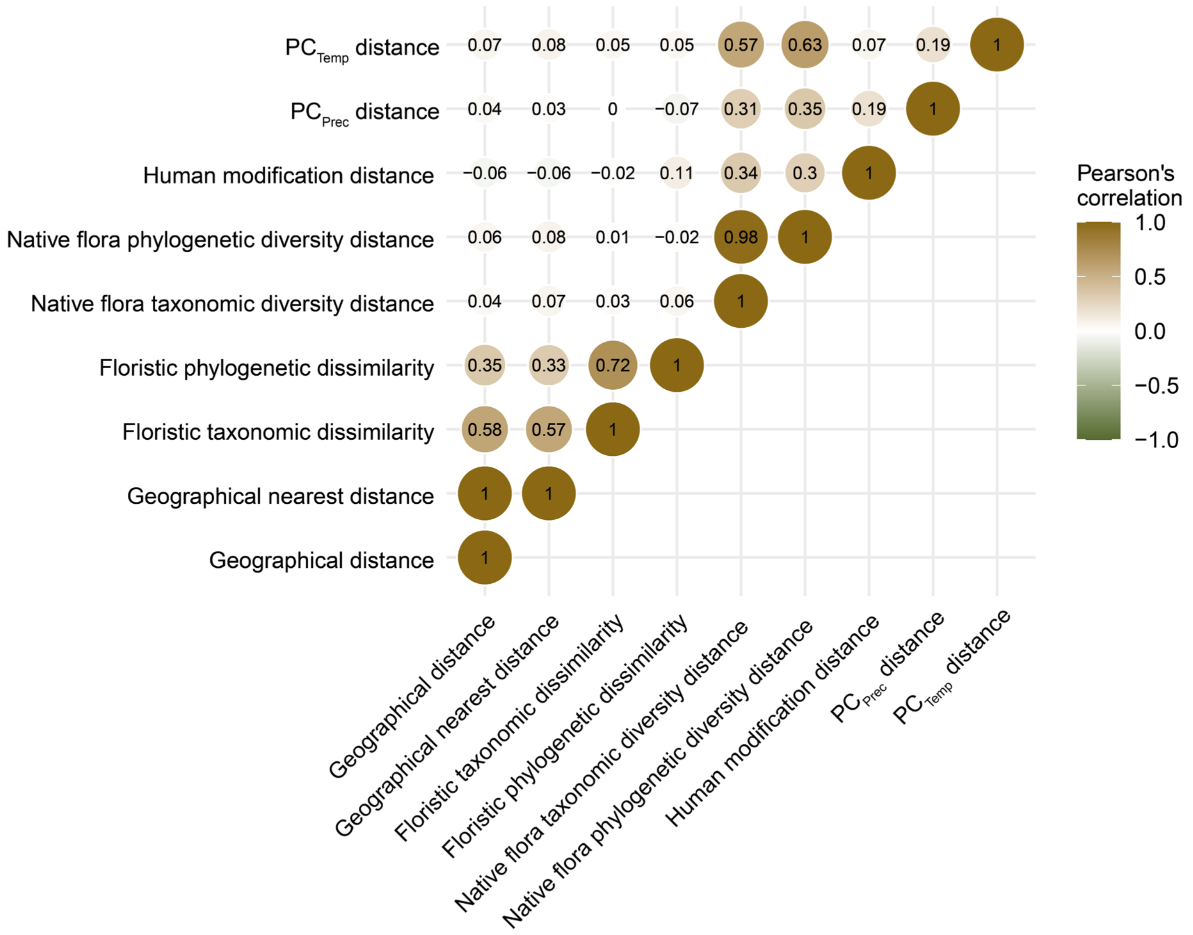

Supplementary Fig. 10 | Correlation plot of various ecological and geographic distances between recipient and donor regions, based on 11,604 alien plant species across 650 regions. The size and color intensity of the circles represent the magnitude of Pearson's correlation coefficients, with darker and larger circles indicating stronger correlations. Source data are provided as a Source Data file.

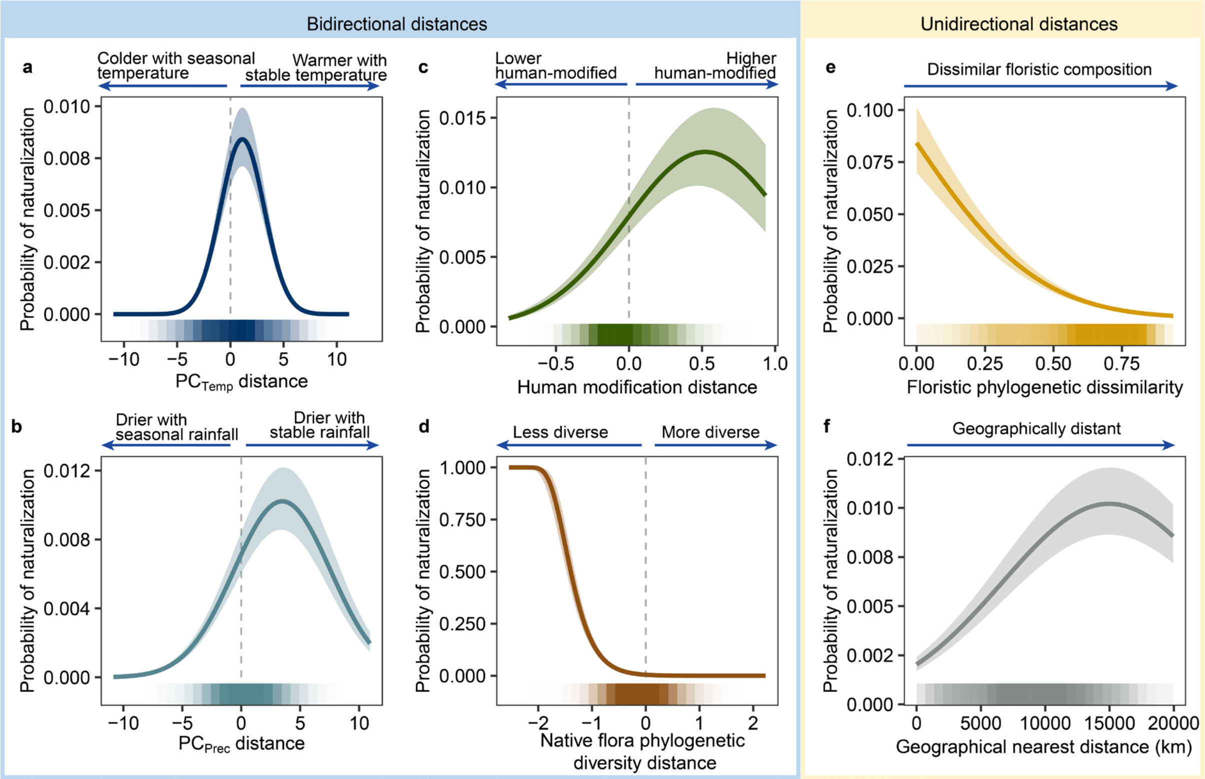

Supplementary Fig. 11 | Partial relationships between naturalization probability and ecological distances between recipient and donor regions, incorporating the geographical nearest distance as a predictor instead of centroid-based geographical distance. The multivariate generalized linear mixed-effects model, based on 6,931,789 observations derived from the naturalization of 11,604 alien plant species across 650 regions, included linear and quadratic terms for each of the six ecological distances (**a**-**f**). The solid lines represent the predicted mean (model fit), and the shaded areas denote the 95% confidence intervals of these predictions. The bar below each plot shows the number of data points in each bin, where the predictor variable was divided into 30 segments; darker shades indicate a higher density of data points within each bin. Note that the y-axis scales differ across panels to reflect variations in data range. Source data are provided as a Source Data file.
