# Supplementary material for: Ecological similarities and dissimilarities between donor and recipient regions shape global plant naturalizations
Source: Nat Commun. 2025 Nov 25;16:10485. doi: 10.1038/s41467-025-65455-y (PMC12647834; doi:10.1038/s41467-025-65455-y)
Supplement: Supplementary file 2 — Reporting Summary [file 41467_2025_65455_MOESM2_ESM.pdf]

nature portfolio

Corresponding author(s): Shao-peng Li
Last updated by author(s): Sep 17, 2025

Reporting Summary

Nature Portfolio wishes to improve the reproducibility of the work that we publish. This form provides structure for consistency and transparency in reporting. For further information on Nature Portfolio policies, see our Editorial Policies and the Editorial Policy Checklist.

Statistics

For all statistical analyses, confirm that the following items are present in the figure legend, table legend, main text, or Methods section.

n/a	Confirmed	
☐	☒	The exact sample size (*n*) for each experimental group/condition, given as a discrete number and unit of measurement
☒	☐	A statement on whether measurements were taken from distinct samples or whether the same sample was measured repeatedly
☐	☒	The statistical test(s) used AND whether they are one- or two-sided<br>*Only common tests should be described solely by name; describe more complex techniques in the Methods section.*
☐	☒	A description of all covariates tested
☒	☐	A description of any assumptions or corrections, such as tests of normality and adjustment for multiple comparisons
☐	☒	A full description of the statistical parameters including central tendency (e.g. means) or other basic estimates (e.g. regression coefficient) AND variation (e.g. standard deviation) or associated estimates of uncertainty (e.g. confidence intervals)
☐	☒	For null hypothesis testing, the test statistic (e.g. *F*, *t*, *r*) with confidence intervals, effect sizes, degrees of freedom and *P* value noted<br>*Give P values as exact values whenever suitable.*
☒	☐	For Bayesian analysis, information on the choice of priors and Markov chain Monte Carlo settings
☒	☐	For hierarchical and complex designs, identification of the appropriate level for tests and full reporting of outcomes
☐	☒	Estimates of effect sizes (e.g. Cohen's *d*, Pearson's *r*), indicating how they were calculated

Our web collection on statistics for biologists contains articles on many of the points above.

Software and code

Policy information about availability of computer code

Data collection	The GloNAF database (Excel format) and the associated shapefiles were obtained from the published data paper. Human modification (HMI) data and WorldClim bioclimatic variables were directly downloaded from their public websites without the use of additional software. The GIFT database was accessed via the GIFT R package (version 1.2.0).
Data analysis	All the data analysis were performed using R version 4.3.3. We used the R package "phytools" (2.1.1) to make the phylogenetic tree. We calculated the phylogenetic metrics using the R package "picante" (1.8.2). We used the R package "normalizer" (0.1.0) to make the bioclimatic variable best approximate a normal distribution. We used the R package "betapart" (1.5.4) to calculate floristic composition's dissimilarities. We used the R package "geosphere" (1.5-14) to calculate geographic distances. We used the R package "ggeffects" (1.5.0) to visualize the predicted relationships for the model. We used the R package "glmm.hp" (0.1-8) to calculate the relative importance of variables. We performed linear mixed-effects models and a binomial generalized linear mixed model by using the R package "lme4" (1.1.28). The code for the analyses and figures are available at figshare (https://doi.org/10.6084/m9.figshare.28513706).

For manuscripts utilizing custom algorithms or software that are central to the research but not yet described in published literature, software must be made available to editors and reviewers. We strongly encourage code deposition in a community repository (e.g. GitHub). See the Nature Portfolio guidelines for submitting code & software for further information.

Data

Policy information about availability of data

All manuscripts must include a data availability statement. This statement should provide the following information, where applicable:

- Accession codes, unique identifiers, or web links for publicly available datasets
- A description of any restrictions on data availability
- For clinical datasets or third party data, please ensure that the statement adheres to our policy

The data generated in this study have been deposited in the figshare (https://doi.org/10.6084/m9.figshare.28513706). Source data are provided with this paper.

Research involving human participants, their data, or biological material

Policy information about studies with human participants or human data. See also policy information about sex, gender (identity/presentation), and sexual orientation and race, ethnicity and racism.

Reporting on sex and gender	Not used in this study
Reporting on race, ethnicity, or other socially relevant groupings	Not used in this study
Population characteristics	Not used in this study
Recruitment	Not used in this study
Ethics oversight	Not used in this study

Note that full information on the approval of the study protocol must also be provided in the manuscript.

Field-specific reporting

Please select the one below that is the best fit for your research. If you are not sure, read the appropriate sections before making your selection.

☐ Life sciences ☐ Behavioural & social sciences ☒ Ecological, evolutionary & environmental sciences

For a reference copy of the document with all sections, see nature.com/documents/nr-reporting-summary-flat.pdf

Ecological, evolutionary & environmental sciences study design

All studies must disclose on these points even when the disclosure is negative.

Study description	Based on the donor and recipient distributions of 11,604 naturalized alien plant species across 650 regions worldwide, we calculated the ecological distances between donor and potential recipient regions for each species. These distances included bidirectional metrics such as temperature, precipitation, human modification, and native plant diversity, as well as unidirectional metrics like phylogenetic composition of local floras and geographic proximity. We further evaluated the relationships between these distances and the probability of naturalization to determine whether alien plants are more likely to naturalize in regions with climates, biological factors, and levels of human modification similar to their donor regions.
Research sample	The distribution data for naturalized alien plants used in this study were sourced from the publicly available GloNAF and GIFT databases, which represent the most comprehensive datasets for naturalized plants and global native plant distributions currently available. Based on these datasets, the plants included in our final analyses comprise all naturalized alien species for which both native and naturalized distribution data were available.
Sampling strategy	From the GloNAF dataset, we identified naturalized alien plant species that had successfully naturalized in at least one region and retained only those with accepted scientific names at the species level. Hybrids were excluded from our analysis, and infraspecific taxa (such as varieties and subspecies) were assigned to the binomial species level. Using the GIFT dataset, we then retrieved native distribution data for these species, creating a global dataset of naturalized alien plants with both native and naturalized distributions.
Data collection	The GloNAF and GIFT databases used in this study were developed by some of the authors. The GloNAF and GIFT data were downloaded by S.Y.F. in October 2023, while the HMI dataset was downloaded in July 2023. Climate datasets were obtained by S.Y.F. in December 2024, and the phylogeny was accessed using the GIFT R package in October 2023.
Timing and spatial scale	The data collection period spanned from July 2023 to December 2024, and all datasets used in this study cover a global spatial scale.
Data exclusions	All naturalized alien plant species with complete native and naturalized distribution data were included in the analysis.
Reproducibility	Our results are reproducible given the data and R code we used are full accessible through Figshare (https://doi.org/10.6084/m9.figshare.28513706).
Randomization	Randomization does not apply as we analyzed existing databases without experiments.

Blinding	Blinding does not apply as we analyzed existing databases without experiments.

Did the study involve field work? ☐ Yes ☒ No

Reporting for specific materials, systems and methods

We require information from authors about some types of materials, experimental systems and methods used in many studies. Here, indicate whether each material, system or method listed is relevant to your study. If you are not sure if a list item applies to your research, read the appropriate section before selecting a response.

Materials & experimental systems

n/a	Involved in the study
☒	☐ Antibodies
☒	☐ Eukaryotic cell lines
☒	☐ Palaeontology and archaeology
☒	☐ Animals and other organisms
☒	☐ Clinical data
☒	☐ Dual use research of concern
☒	☐ Plants

Methods

n/a	Involved in the study
☒	☐ ChIP-seq
☒	☐ Flow cytometry
☒	☐ MRI-based neuroimaging

Plants

Seed stocks	Not used in this study
Novel plant genotypes	Not used in this study
Authentication	Not used in this study
